# Effect of Interfacial SiO_*x*_ Defects on the Functional Properties of Si-Transition Metal Oxide Photoanodes for Water Splitting

**DOI:** 10.1021/acsami.3c09555

**Published:** 2023-10-02

**Authors:** P. Ragonese, B. Kalinic, L. Franco, L. Girardi, B. M. Fernández Peréz, D. Carbonera, G. Mattei, G.-A. Rizzi, C. Maurizio

**Affiliations:** † Physics and Astronomy Department, University of Padova, Via Marzolo 8, Padova I-35131, Italy; ‡ Department of Chemical Sciences, University of Padova, Via Marzolo 1, Padova I-35131, Italy

**Keywords:** water splitting, Si-based photoanodes, cobalt oxide, native silicon oxide, surface states, electron paramagnetic resonance, charge transfer

## Abstract

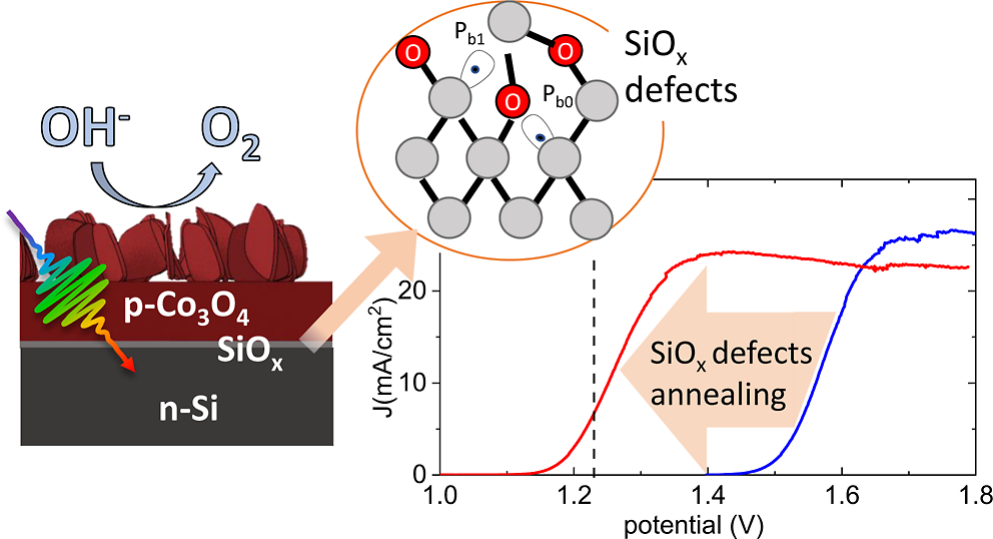

The transfer of photogenerated charges through interfaces in heterojunction photoanodes is a key process that controls the efficiency of solar water splitting. Considering Co_3_O_4_/SiO_*x*_/Si photoanodes prepared by physical vapor deposition as a representative case study, it is shown that defects normally present in the native SiO_*x*_ layer dramatically affect the onset of the photocurrent. Electron paramagnetic resonance indicates that the signal of defects located in dangling bonds of trivalent Si atoms at the Si/SiO_*x*_ interface vanishes upon vacuum annealing at 850 °C. Correspondingly, the photovoltage of the photoanode increases to ≈500 mV. Similar results are obtained for NiO/SiO_*x*_/Si photoanodes. Photoelectrochemical analysis and impedance spectroscopy (in solution and in the solid state) indicate how the defect annealing modifies the Co_3_O_4_/SiO_*x*_/Si junction. This work shows that defect annealing at the solid–solid interface in composite photoanodes strongly improves the efficiency of charge transfer through interfaces, which is the basis for effective solar-to-chemical energy conversion.

## 1 Introduction

Solar-assisted water splitting is a promising route to convert solar energy into chemical energy through green hydrogen generation by water splitting.^1^ Si-based photoanodes, due to the good spectral match between the Si absorption spectrum and the solar emission and to the high mobility and long lifetime of the photogenerated charges, offer an effective solution to speed up the slow water oxidation reaction, constituting the bottleneck of the whole process.^2^ To cope with the low intrinsic catalytic activity and durability of Si in the harsh conditions typically used to favor water splitting (pH = 14), different strategies have been planned, the most promising of which is the surface functionalization with metal^3−7^ or semiconductor nanostructures^8−10^ that can act both as a protective layer and as a catalyst for the oxygen evolution reaction. Moreover, proper engineering of the local electric field at the n-Si/catalyst interface can boost the effectiveness of the charge separation and transfer toward the catalyst/solution interface. To this purpose, in some cases, a p^+^n-Si buried junction is included in the fabrication,^9,11−13^ with the result that a higher photovoltage is obtained, together with a generally increased complexity of the system. Typically, the Si/catalyst interface has been deeply studied in the case of a Schottky junction, i.e., in the case of a metallic catalyst,^3,7,14,15^ while the experimental investigations on the Si-transition metal oxide (TMO) photoanodes, that always include a thin SiO_*x*_ interface layer, have been mainly focused on the TMO phase and electronic band structure.^10,16−18^ Nevertheless, the Si-TMO solid–solid interface can, in principle, strongly act on the charge transfer effectiveness. Defects at the solid–solid interface can constitute recombination centers or can host localized charges, both detrimental to the charge transport efficiency.^7,12^ Their presence in a few-atom thick interface is hardly directly detectable, so that the defect analysis is often referred to the whole material rather than to the interface between two materials.^19−21^ Many different point defects at the Si/SiO_*x*_ interface have been extensively studied in the past by means of electron paramagnetic resonance (EPR) spectroscopy, and several types of paramagnetic defects have been identified, either at the surface or in the bulk of the oxide layer.^22,23^ Some defects have been recognized as a source of charge trapping and charge recombination and are therefore responsible for performance instability or even failure in a variety of microelectronic devices based on Si/SiO_*x*_ interfaces.

In this work, starting from a n-Si/SiO_*x*_/Co_3_O_4_ composite heterojunction that is well-known to efficiently work for solar-assisted water splitting, it is shown that paramagnetic silica defects, in the form of dangling bonds of trivalent Si atoms, have a relevant effect on the measured photovoltage. Without any other action on the composite system, the simple thermal annealing of these defects prior to the TMO overlayer deposition causes an increased photovoltage to about 500 mV. The experimental results indicate that this is related to an optimized interface where the built-in potential localized at the n-Si/SiO_*x*_/Co_3_O_4_ junction favors the separation of the photogenerated charges, which is highly desirable for an efficient oxygen evolution reaction.

## 2 Results and Discussion

### 2.1 Photoelectrochemical Characterization

**Figure 1:**
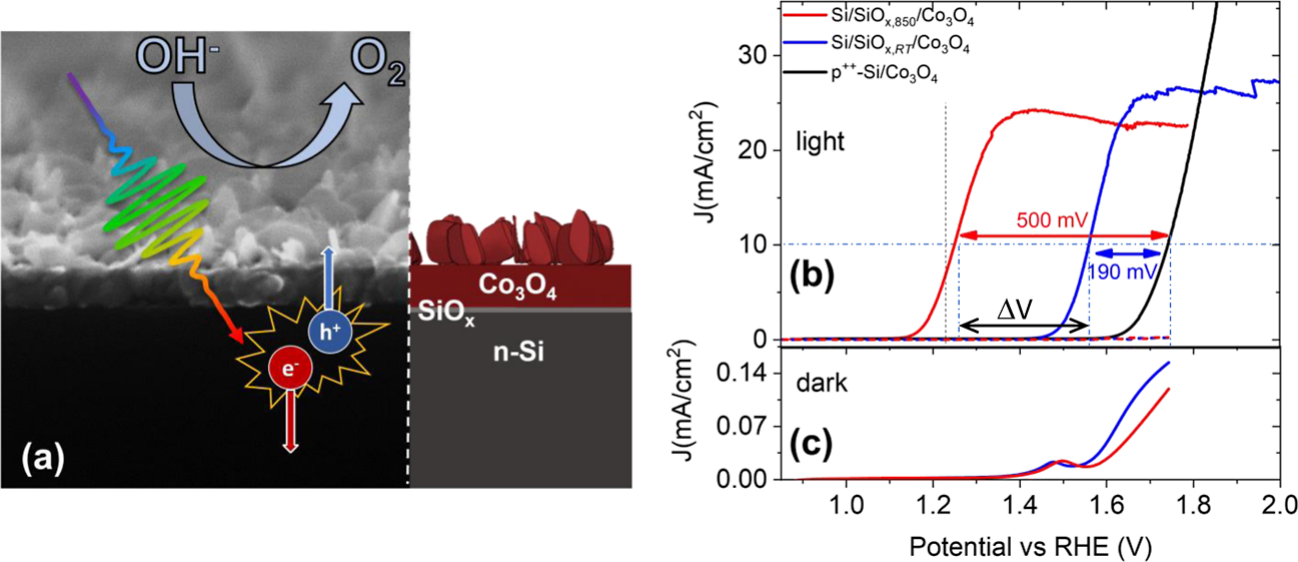
(a) SEM image (tilted view) and a sketch of the Si/SiO_x_/Co_3_O_4_ nanopetals photoanode. The thickness of the Co_3_O_4_ layer is approximately 50 nm. (b,c) Current–voltage curves of the n-Si/SiO_*x*_/Co_3_O_4_ photoanodes recorded under 1.5 AM solar illumination (b) and in the dark conditions (c) in KOH solution (pH 13.8) with (red) or without (blue), including an annealing step of the substrate prior to the cobalt deposition. The current–voltage curve recorded for the reference p^++^-Si/SiO_*x*_/Co_3_O_4_ anode is also reported in (b) for comparison, and the reference voltage for water oxidation is marked. The photovoltage for the two photoanodes is marked.

**Figure 2:**
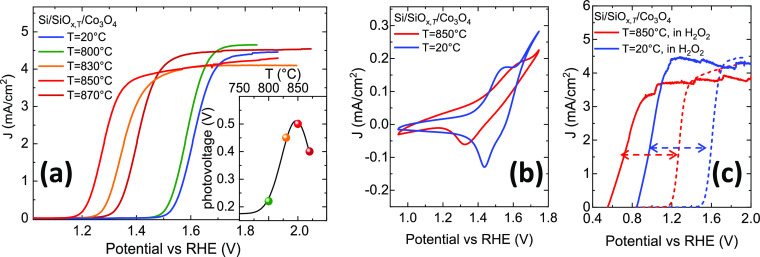
(a) Current–voltage curves of the n-Si/SiO_*x*_/Co_3_O_4_ photoanodes annealed in vacuum before Co deposition at selected temperatures, recorded under 25 mW/cm^2^ illumination by a white LED in KOH solution (pH = 13.8). Inset: photovoltage as a function of the substrate annealing temperature; the solid line is a guideline to the eye. (b) Cyclic voltammetry curves recorded for the two (extreme) photoanodes in (a). (c) Current–voltage curves of the n-Si/SiO_*x*_/Co_3_O_4_ photoanodes recorded under illumination (white LED) in 1 M KOH solution +0.1 M H_2_O_2_ with (red) or without (blue), including the annealing step of the substrate before the cobalt deposition. The corresponding curves measured in a 1 M KOH solution are also reported for comparison (dashed lines).

n-Si/SiO_*x*_/Co_3_O_4_ photoanodes have been prepared by physical vapor deposition followed by oxidizing annealing, as described elsewhere.^24^ The last thermal oxidation step promotes the formation of Co_3_O_4_ nanopetals (see Figure 1a and S1a) that increase the electrode surface in contact with the solution, which is beneficial for catalytic processes occurring at the photoanode surface.^24^Figure 1b reports the current–voltage curves recorded upon AM 1.5 solar illumination in KOH (pH = 13.8) for two Si/SiO_*x*_/Co_3_O_4_ photoanodes. In both cases, a Co metallic layer was deposited on Si/SiO_*x*_ with the native SiO_*x*_ layer grown at room temperature (Si/SiO_*x*,RT_) after HF etching by a standard RCA SC-2 procedure.^25^ The difference is that, in the case of Si/SiO_*x*,850_/Co_3_O_4_, the substrate underwent an additional vacuum annealing (*p* = 5 × 10^–5^ mbar) at 850 °C prior to Co deposition (Si/SiO_*x*,850_). The two curves are compared with the corresponding electrocatalytic response of a p^++^-Si/Co_3_O_4_ anode prepared in a similar way (whose current onset^17^ and Tafel slope^26^ are similar to literature results (Figure S1b). It is evident from the remarkable effect of the predeposition annealing that cathodically shifts the photocurrent onset of Δ*V* ≈ 300 mV, i.e., below the thermodynamic water oxidation potential. Considering a reference current of 10 mA/cm^2^, the photovoltage is about 190 mV for the Si/SiO_*x*,RT_/Co_3_O_4_ photoelectrode and about 500 mV for the Si/SiO_*x*,850_/Co_3_O_4_ one (Figure 1b). This observed difference between the two photoanodes is highly reproducible, as reported in Figure S2, where the results on several photoanodes prepared in the same way are compared. Both Si/SiO_*x*_/Co_3_O_4_ photoanodes show a maximum photocurrent density of about 25–28 mA/cm^2^ (Figure 1b), comparable with the results on similar systems.^10,11^ Since the two photoanodes are obtained with the same Co deposition and the same postdeposition annealing treatment and the two Co-oxide nanopetal layers are the same when investigated by X-ray diffraction (XRD), X-ray photoelectron spectroscopy (XPS), and scanning electron microscopy (SEM), the origin for the different behavior has to be found in the different Si/SiO_*x*_/Co_3_O_4_ solid–solid interface. To better investigate this point, a set of photoanodes was prepared, whose only difference is the thermal annealing in a vacuum at a defined temperature of the Si/SiO_*x*_ substrate (SiO_*x*,*T*_). Figure 2a reports the corresponding current–voltage curves. While thermal annealing at 800 °C of the substrate (before Co deposition) only slightly improves the photovoltage of the Si/SiO_*x*_/Co_3_O_4_ photoanode, for substrate annealing treatments in the range 830–850 °C, a significant and progressive increase in the photovoltage is observed. It is known that at high temperatures in vacuum, the Si/SiO_*x*_ surface is unstable and undergoes etching through the formation of volatile SiO.^27^ In our case, this phenomenon severely damaged the Si surface at 900 °C, and it appeared visible in some spots already at 870 °C. Correspondingly, the obtained photovoltage upon 870 °C annealing is lower than that upon 850 °C annealing (see also the inset in Figure 2a). The cyclic voltammetry curves for the two extreme cases (Si/SiO_*x*,RT_/Co_3_O_4_ and Si/SiO_*x*,850_/Co_3_O_4_) in dark conditions are reported in Figure 2b. For the case of the Si/SiO_*x*,RT_/Co_3_O_4_ photoanode, both the Co^2+^/Co^3+^ redox peaks (the anodic peak at about 1.45 V and the cathodic peak at about 1.55 V vs RHE) are visible. For the case of the Si/SiO_*x*,850_/Co_3_O_4_ photoanode, an increased voltage difference between the cathodic and anodic peaks is found, suggesting a lower charge transfer rate in dark conditions toward/from the solution.^28^

To clarify if the remarkable difference in the measured photovoltage is related to a more efficient charge transfer occurring at the solid–solution interface or at the solid–solid interface, a current–voltage curve has been recorded for the two photoelectrodes in the presence of H_2_O_2_, acting as a hole scavenger. The results, reported in Figure 2c, show that the two current–voltage curves are both cathodically shifted of about 650 mV with respect to the same curve recorded in KOH solution, in agreement with the different oxidation potentials of H_2_O_2_ and H_2_O. Still, the Si/SiO_*x*,850_/Co_3_O_4_ photoanode maintains the best photoelectrocatalytic performance with respect to the Si/SiO_*x*,RT_/Co_3_O_4_ one, showing that the improvement is related to the optimized solid–solid interface.

It is interesting to note that very similar results (increased photovoltage upon Si/SiO_*x*_ substrate annealing at 850 °C) are also obtained in the case of (i) Si/SiO_*x*_/NiO photoanodes (Si/SiO_*x*,RT_/NiO and Si/SiO_*x*,850_/NiO), obtained upon metal deposition of a Ni film and thermal oxidation (Figure S3), and (ii) Si/SiO_*x*_/Co_3_O_4_ photoanodes obtained upon reactive physical vapor deposition of Co in an oxygen atmosphere plus thermal oxidation (Figure S4). In all the cases, the onset of the photocurrent is at lower potential values for the Si/SiO_*x*_ substrate annealed at 850 °C in vacuum.

### 2.2 Si/SiO_*x*_ Characterization

To gain information on the surface layer of the Si/SiO_*x*,RT_ and Si/SiO_*x*,850_ substrates before the metal deposition, an X-ray photoelectron spectroscopy experiment has been performed.

**Figure 3:**
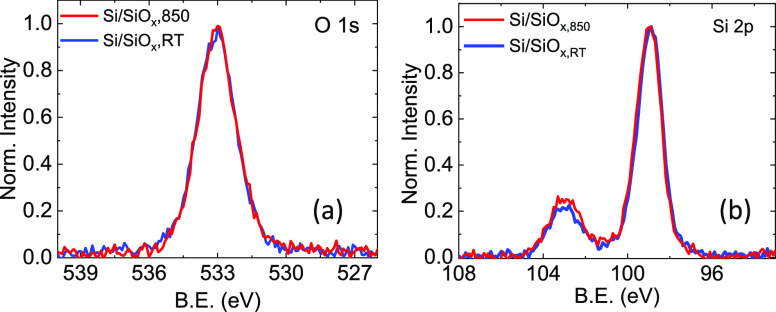
XPS spectra in the (a) O 1s and (b) Si 2p electronic energy range signals for the Si/SiO_*x*,850_ and Si/SiO_*x*,RT_ substrates.

**Figure 4:**
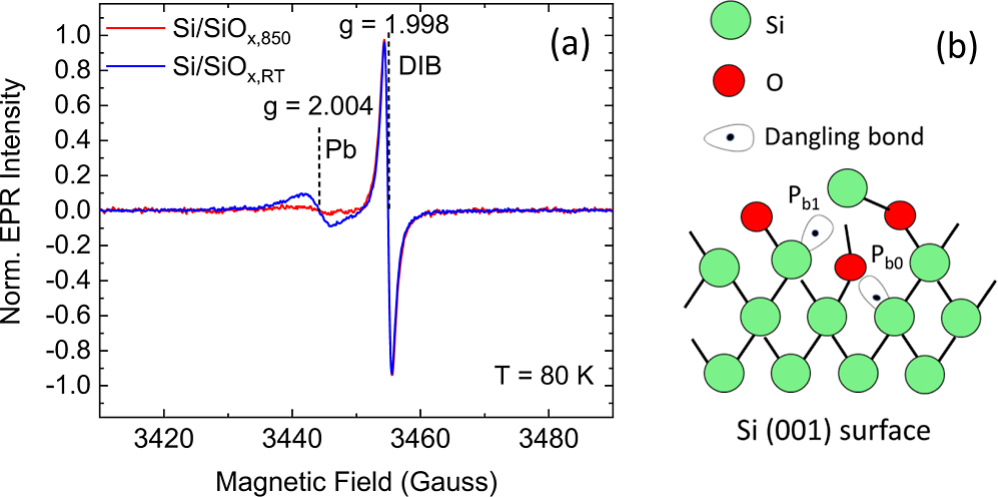
(a) EPR spectra of Si/SiO_*x*,RT_ (blue line) and of Si/SiO_*x*,850_ (red line) samples recorded at *T* = 80 K. The two spectra are normalized to the maximum of the *g* = 1.998 line. Each spectrum is the average over different samples. (b) Sketch of the structure of the P_*b*_ defects.

In Figure 3, the XPS spectra reporting the O 1s and Si 2p signals are shown for the two substrates. The O 1s signal at about 532.7 eV mainly takes a contribution from the O atoms in the SiO_*x*_ native layer. As expected, the Si 2p signal contains two well-resolved components at about 99 and 103 eV, assigned respectively to Si(0) in the bulk and Si(IV) in the SiO_*x*_ oxide overlayer. It is known that the Si/SiO_*x*_ layer at the interface consists of two regions.^29^ The region closer to the free surface contains Si(IV), while the near-interface region contains few atomic layers of Si atoms in intermediate oxidation states, i.e., Si(I), Si(II), and Si(III) with binding energy values between 100 and 101.5 eV.^29^ In our case, only a minor fraction of Si with an intermediate oxidation state is detected (see Figure S5). From the XPS analysis of the peak intensities, the O/Si ratio resulted the same within the experimental uncertainty for both substrates. Moreover, it is evident that the thickness of the silicon oxide layer is also practically identical for the two substrates, being the Si(0)/Si(IV) ratio the same within the experimental uncertainty, in agreement with spectroscopic ellipsometry measurements (not reported) that indicate in both cases a ≈1.5 nm thick oxide layer. To gain more information about the possible presence of point defects at the Si/SiO_*x*_ interface, which are known to affect the charge transport across the interfaces, we used EPR spectroscopy. The goal was to identify the presence of paramagnetic point defects at the Si/SiO_*x*_ interface and to check whether the annealing step was able to modify the number and properties of such point defects. In order to avoid the spectral interference from Co_3_O_4_ and to focus on the effects of the annealing process, we measured the Si/SiO_*x*_ substrates before and after thermal annealing and without the Co_3_O_4_ layer. In the two substrates, at room temperature, only an extremely weak EPR spectrum is detected (not reported), showing a line approximately at *g* = 2.004, barely emerging from noise even after prolonged acquisition. However, at lower temperature (*T* = 80 K), a much stronger spectrum is obtained in both cases, as reported in Figure 4.

The EPR spectrum of the Si/SiO_*x*,RT_ substrate is composed of two lines. At a lower magnetic field, there is a broad line centered at a *g*-factor of about 2.004 with approximately a Gaussian line shape and a line width of 7 G. At higher field, there is a narrow line centered at *g* = 1.998 with a Lorentzian shape and a line width of about 1.5 G. Both EPR lines are not saturated at the microwave power used (≤10 mW). The line at *g* = 1.998 is assigned to the donor-impurity band (DIB) in phosphorus-doped silicon (n-Si).^30^ This EPR line is described in the literature as extremely broad and weak at room temperature (and therefore correctly not detected in our spectra at 298 K), but narrow and more intense at temperatures down to about 50 K. The presence of this line turned out to be useful as a *g*-factor and intensity reference, as described in the following. The band at the lower field (*g* = 2.004) can be assigned, on the basis of the *g*-factor, to Si/SiO_*x*_ interface defects, labeled in the literature as Pb_0_ and Pb_1_ that are formed at the Si(100)/SiO_2_ interface. They are attributed to unpaired electrons located in a dangling bond of trivalent silicon, as sketched in Figure 4b.^23^ The *g*-factor tensors reported in the literature for the Pb_0_ and Pb_1_ centers are axially symmetric or slightly rhombic and their principal values, although not uniform in the literature, are *g*_∥_ = 2.0015 and *g*_⊥_ = 2.0087. Our spectral line, centered at *g* = 2.004, probably originated from the unresolved overlap of different lines belonging to different defects (in the following labeled P_*b*_). The two bands in the EPR spectrum for the Si/SiO_*x*,RT_ substrate have approximately the same (doubly integrated) intensity. Assuming that the nominal number density of the phosphorus centers in the n-Si wafer is 10^15^ cm^–3^, and considering the dimensions of the measured samples, an approximate estimate of the P_*b*_ defect densities can be obtained. The resulting defect density on the surface of the Si wafer is calculated to be ≈1 × 10^13^ defects/cm^2^ for the Si/SiO_*x*,RT_ sample, consistent with the values previously reported.^31^ Considering that a ≈1.5 nm SiO_2_ layer contains ≈3.3 × 10^15^ Si/cm^2^ ( = 6.6 × 10^22^ at./cm^3^), less than 1% of Si atoms in the oxide are affected by these defects. This also indicates that these defects are below the detection limit for the XPS experiment, consistent with the obtained results. The intensity of the *g* = 2.004 line is strongly reduced after vacuum annealing at 850 °C, as shown in Figure 4. The effect of thermal treatment is therefore the almost complete elimination of the species, giving rise to the EPR line at *g* = 2.004, whereas the line at *g* = 1.998 is unaffected. The annealing therefore induced a valence saturation, i.e., a pairing of unpaired electrons in the trivalent silicon dangling bonds. In this way, the paramagnetic Pb_0_ and Pb_1_ defects are changed into diamagnetic species, which are not detected by EPR. The presence of dangling bond defects has been demonstrated in previous reports to be related to the adverse electrical and charge transport behavior of the devices based on Si/SiO_*x*_ interfaces.^32,33^ The reduction of the defect density is, therefore, necessary to avoid such decreased device performances. Our evidence from EPR spectra unequivocally indicates that the vacuum annealing we performed can significantly reduce the number of dangling bonds present in the unannealed Si/SiO_*x*_ sample.

Considering that, apart from the SiO_*x*_ interface, the Si/SiO_*x*,850_/Co_3_O_4_ and Si/SiO_*x*,RT_/Co_3_O_4_ photoanodes are identical, we can reasonably indicate that the lower photovoltage observed for the Si/SiO_*x*,RT_/Co_3_O_4_ photoanode is related to the higher density of SiO_*x*_ defects, which are known to modify the band bending at the interface between two semiconductors.

### 2.3 Heterojunction Characterization

**Figure 5:**
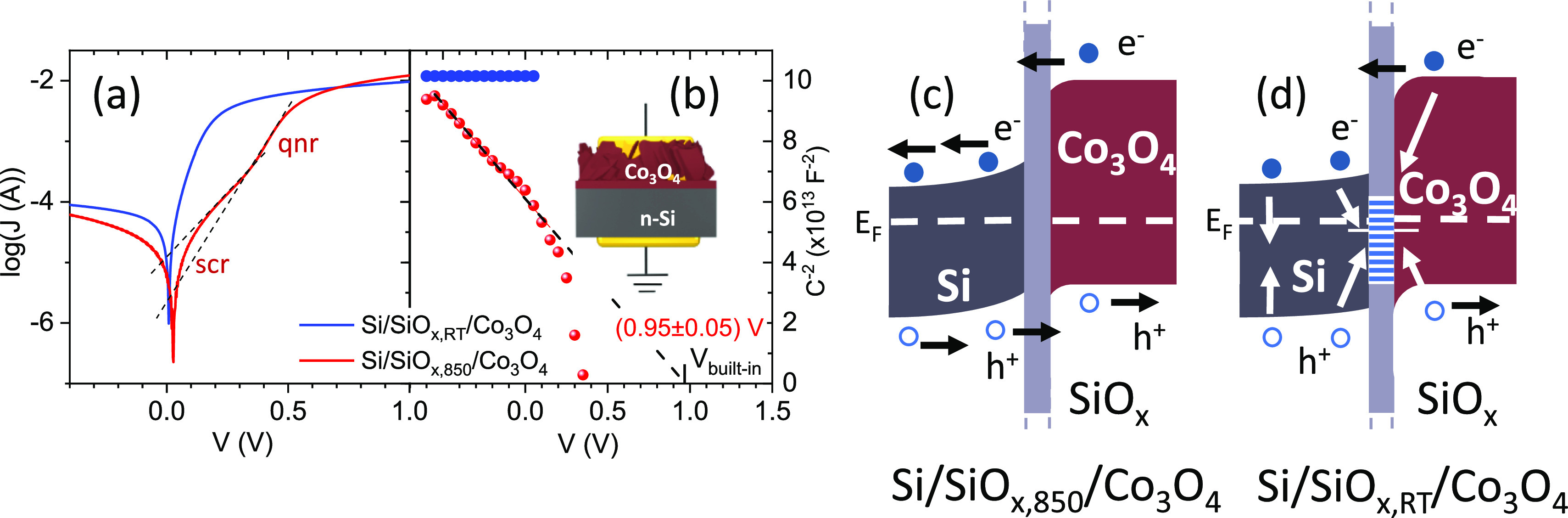
(a) Solid-state current–voltage curves measured for the two cases where the silicon wafer was (Si/SiO_*x*,850_/Co_3_O_4_, red line) or was not (Si/SiO_*x*,RT_/Co_3_O_4_, blue line) annealed before Co deposition. The red curve exhibits the typical feature of a p–n junction with a space charge region (scr) and a quasi-neutral region (qnr). (b) Mott–Schottky analysis obtained from electronic impedance spectroscopy [same color code as in (a)]. (c,d) Sketch of the band structure in the two cases. Black arrows indicate the motion of the photogenerated charges. In (d), trap states induced by SiO_*x*_ defects are sketched (Si/SiO_*x*,RT_/Co_3_O_4_ case) with their neutral energy level below the Fermi energy E_*F*_. They result in an additional barrier (Δ*V* ≈ 300 mV, see Figure 1b) for the photogenerated charges. White arrows indicate the increased recombination probability of the photogenerated charges.

To investigate the electronic properties of the Si/SiO_*x*_/Co_3_O_4_ junction, Cr–Au ohmic contaminants were deposited on both sides of the junction. The current–voltage curves measured for both cases are shown in Figure 5a. Both junctions show a rectifying behavior, visible from the lower current recorded at negative voltage (inverse bias), as expected for the n-Si/p-Co_3_O_4_ junction. The Si/SiO_*x*,850_/Co_3_O_4_ is characterized by a lower dark current at inverse potential and by the typical features of a p–n junction, with a space charge region (*V* = 0–0.5 V) and a quasi-neutral region (*V* = 0.4–0.6 V), both present and well distinguishable. This curve can be described by considering two diodes related to the two voltage ranges. In particular, from the two linear ranges in the log–lin plot of Figure 5a, good quality factors of a few units (see Supporting Information) are obtained,^34^ more than reasonable considering the simplicity of the used fabrication process. In this case, the space charge region induces a built-in potential on the junction that, upon inverse polarization and illumination, favors the separation of the photogenerated charge couples. The case where the substrate was not annealed before Co deposition also shows a rectifying behavior but with a higher inverse current and a less pronounced space charge region. This is in agreement with the significantly lower photovoltage measured in the photoelectrochemical experiment.

Solid-state electronic impedance spectroscopy allows one to measure the frequency response of the junction. The data can be fitted on the basis of an RC circuit. The corresponding Mott–Schottky plot that results from the capacitance values is shown in Figure 5b. In the case of the Si/SiO_*x*,850_/Co_3_O_4_ photoanode, a built-in potential of 0.95 V is obtained. From the measured energy gap of the Co_3_O_4_ layer (see the Tauc plot in Figure S7) and the Fermi energy of Si (determined from the doping condition), the band structure of the system (Si/SiO_*x*,850_/Co_3_O_4_) can be sketched as in Figure 5c. In this case, in particular, the built-in potential is the energy barrier that prevents the electronic flow from n-Si to p-Co_3_O_4_. From the reported sketch, the Fermi energy level for Co_3_O_4_ is found to be about 0.63 eV above the top of the valence band, in agreement with the value previously measured on a similar system.^24^ If the annealing before the Co deposition is omitted, the equivalent capacitance obtained (in the condition of inverse bias) does not vary with the applied potential, indicating that the electronic behavior of the junction is more dependent on the defects at the solid–solid interface rather than on the applied potential.^35,36^ This phenomenon is analogous to the Fermi level pinning for a metal–semiconductor junction.

So, the almost rigid shift toward higher voltage values of the Si/SiO_*x*,RT_/Co_3_O_4_ photoelectrochemical current–voltage curve (Figure 1b) with respect to the Si/SiO_*x*,850_/Co_3_O_4_ case (see Figure 1b), together with the EPR results and the solid state current–voltage and impedance spectroscopy analyses, suggest that the defects at the solid–solid interface of the Si/SiO_*x*,RT_/Co_3_O_4_ photoanode might indeed host electric charges, inducing an additional electric field. This phenomenon affects the transfer of the photogenerated charges through the solid–solid interface and modifies the depletion region of the junction. In particular, localized interface states can host negative charges if their neutral energy level is lower than the Fermi energy.^36^ In this case, the electric field associated with these charges tends to bend the Si and Co_3_O_4_ electronic bands downward, negatively affecting the photovoltage of the photoanode in a way that is compatible with the observed reduced performances (see Figure 5d). Considering that these defects shift anodically the onset of the photocurrent of Δ*V* ≈ 300 mV (Figure 1b) and in the hypothesis that the depletion region is mostly extended on the Si side, the effective surface charge σ could be roughly estimated as , where ϵ and λ_D_ are the dielectric constant (≃11.7) and the Debye length in Si, respectively (λ_D_ ≈ 100 nm considering the doping condition). It is obtained σ ≈ 4 × 10^11^ charges/cm^2^, corresponding to about 3 elementary charges for every hundred defects. That is, starting from the detection of P_*b*_ neutral defects by EPR, our results suggest that an effective charge is nested during subsequent Co deposition and annealing. This can also account for the modification of the Mott–Schottky plot, as observed for the Si/SiO_*x*,RT_/Co_3_O_4_ photoanode.

**Figure 6:**
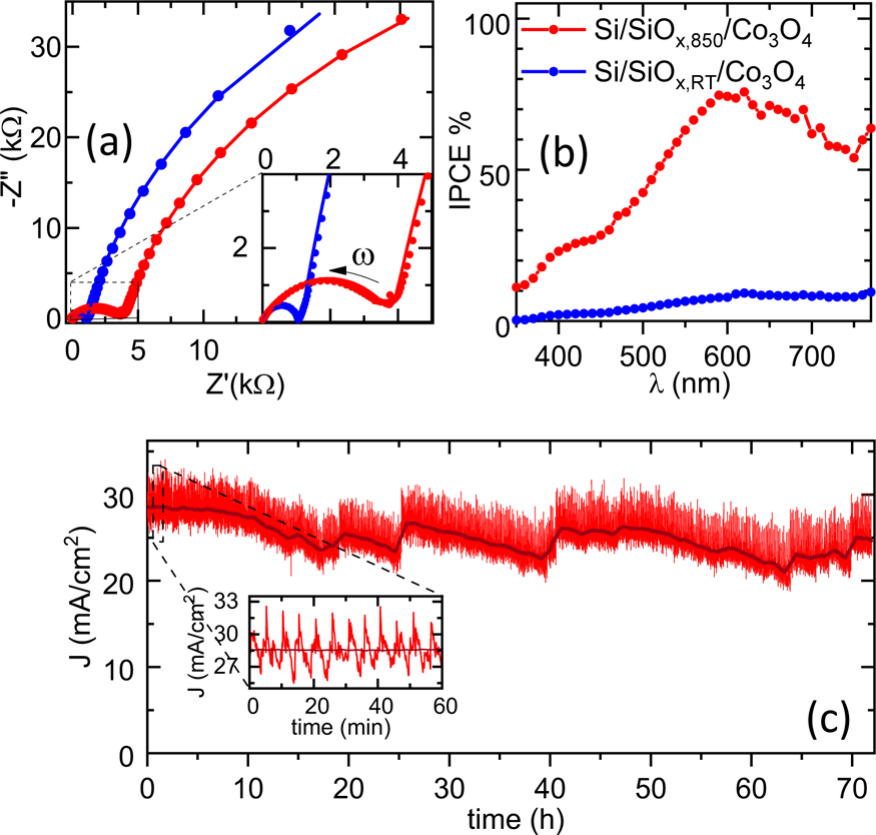
(a) Electrochemical impedance spectra (markers) measured at the open circuit potential in dark condition with best–fit curves (solid line) for Si/SiO_*x*,850_/Co_3_O_4_ (red) and Si/SiO_*x*,RT_/Co_3_O_4_ (blue) photoanodes. (b) Incidence photon-to-current conversion efficiency recorded at *V* = 1.5 V vs RHE for the photoanodes in (a). (c) Chronoamperometric curve measured in saturation conditions for the Si/SiO_*x*,850_/Co_3_O_4_ photoanode. The inset shows the fluctuations of the current due to the evolution of bubbles.

Further insights come from the analysis of the frequency response of the photoanodes measured under their working conditions. Figure 6a reports electrochemical impedance spectroscopy (EIS) data recorded under dark conditions at the open circuit potential (OCP) for the Si/SiO_*x*,850_/Co_3_O_4_ and Si/SiO_*x*,RT_/Co_3_O_4_ anodes. When looking at the imaginary versus real part of the impedance, in both cases, two arcs are visible. The largest one is recorded in the low-frequency range (from 0.1 to 10 Hz). It evidence a high impedance because the charge transfer at the anode-solution interface is not active at the OCP. The smaller arc is recorded in the high-frequency range (maximum of −Z″ at about 2.5 kHz, see inset of Figure 6a) and is related to the charge transfer through the solid–solid interface of the heterojunction.^37^ The analysis of the impedance spectra (details in the Supporting Information) shows that the solid–solid interface for the photoanode with the annealed substrate is significantly more resistive than for the photoanode with the optimized interface. This is in agreement with the low concentration of defects at the solid–solid interface of Si/SiO_*x*,850_/Co_3_O_4_, which hinders the current flow in dark conditions, resulting in a higher impedance with respect to the Si/SiO_*x*,*RT*_/Co_3_O_4_ anode. A high-resistivity behavior is normal for an inversely polarized p–n junction when not illuminated. We note that SiO_*x*_ interfacial defects remarkably affect the onset of the photocurrent because they locally modify the electric field of the inverse-polarized p–n junction. If the TMO layer were the only photoactive material, then the photoanode would work only with a substrate-TMO interface, forming either a direct junction or an ohmic contact. In this case, the quality of the substrate/TMO interface layer would be less relevant.^38^

### 2.4 Photoanode Efficiency and Durability

In Figure 6b, the incident photon-to current conversion efficiency spectra are reported for the Si/SiO_*x*,850_/Co_3_O_4_ and Si/SiO_*x*,*RT*_/Co_3_O_4_ photoanodes, recorded at *V* = 1.5 V vs RHE. For the Si/SiO_*x*,850_/Co_3_O_4_ photoanode (that is in saturation conditions, see Figure 1b), a maximum photon-to-current conversion efficiency of about 80% is achieved at λ ≈ 600 nm, that well matches the range of maximum solar irradiance, and that is well comparable with previous results.^17^ The Si/SiO_*x*,RT_/Co_3_O_4_ photoanode exhibits a lower efficiency in the whole spectral range, in agreement with the lower photocurrent measured at the same polarization potential (Figure 1b). In Figure 6c, the chronoamperometric curve recorded for the Si/SiO_*x*,850_/Co_3_O_4_ photoanode in saturation conditions is shown. The bubble formation in the solution leads to a large oscillation of the photocurrent. Nevertheless, the photoanode shows stable behavior over several tens of hours.

Overall, these results show how paramagnetic defects, in the form of dangling bonds in trivalent Si at the n-Si/SiO_*x*_ interface, dramatically affect the photovoltage of the Si-TMO photoanodes. Proper annealing of the SiO_*x*_ paramagnetic defects significantly improved the electrical properties and the built-in potential of the p–n junction formed with a layer of a p-type TMO. This results in a high photovoltage that is comparable to the one obtained with similar junctions based on more complex synthesis techniques, such as atomic layer deposition, possibly coupled with buried p^+^n junctions.^10,11,17^

## 3 Conclusions

The functional properties of n-Si/SiO_*x*_/Co_3_O_4_ photoanodes prepared by physical vapor deposition have been investigated for water splitting and related to the presence of defects at the Si/SiO_*x*_ interface. In particular, SiO_*x*_ defects in the form of unpaired electrons in trivalent Si dangling bonds could be detected by EPR, which also shows that they can be annealed by proper thermal treatment in vacuum. n-Si/SiO_*x*_/Co_3_O_4_ photoanodes prepared after defect annealing exhibit a ≈500 mV photovoltage, i.e., about 300 mV higher than the unannealed case. An increased photovoltage upon defect annealing is also observed when NiO is used instead of Co_3_O_4_. The results of the experiments indicate that defects act on the Si depletion region, unfavorably lowering the bending of the electronic energy bands, with the result that an additional voltage supply is required to promote the photoelectrochemical water splitting. Under 1 sun illumination, the optimized photoanode shows about 8 mA/cm^2^ at the water oxidation potential and a saturation current of about 28 mA/cm^2^, with stability in working condition over several days (≈10% of efficiency loss in 70 h). This work shows that defect annealing at the solid–solid interface strongly improves the effectiveness of charge transfer through interfaces, which is the basis for effective solar-to-chemical energy conversion.

## 4 Experimental Section

Phosphorus-doped Si wafers (100-oriented, resistivity = 1–10 Ω cm) were cut into 1 × 1 cm^2^ pieces and cleaned in a piranha solution (H_2_SO_4_/H_2_O_2_ = 3:1) for 30 min at 60 °C. The native silicon oxide layer was then removed through an 5% HF dip for 1 min. Then, they were immersed in a RCA solution (HCl/H_2_O_2_/H_2_O = 1:1:5) for 10 min at 75 °C, rinsed with Milli-Q water, and dried in N_2_ (Si/SiO_*x*,RT_). After the RCA cleaning procedure, part of the substrates were annealed under vacuum at 850 °C for 2 h (Si/SiO_*x*,850_). The base pressure for the vacuum annealing was *P* = 2 × 10^–5^ mbar. Atomic force microscopy (AFM, NT-MDT instrument) was used to probe the surface morphology of the obtained substrates (Figure S10).

For the EPR measurements, slides of 2 × 25 mm size were cut from the Si/SiO_*x*,850_ and Si/SiO_*x*,RT_ substrates and inserted into quartz tubes filled with N_2_ gas. The EPR spectra were recorded with a Bruker Elexsys580 spectrometer at X-band (9–10 GHz) using an ER4118X-MD5 dielectric ring resonator inserted into an ER 4118CF cryostat for low-temperature measurements. All the spectra have been recorded at the temperature of 80 K using the following parameters: microwave power 10 mW, field modulation amplitude 0.5 G, magnetic field sweep 100 G, 100 scans.

XPS was performed in a custom-built ultrahigh vacuum (UHV) chamber at a base pressure of 5 × 10^–10^ mbar. A nonmonochromatized double-anode X-ray source (Omicron DAR-400, Scienta-Omicron GmbH, Uppsala, Sweden), a hemispherical electron analyzer (Omicron DAR-400, Scienta-Omicron GmbH, Uppsala, Sweden), and a 5 channel electron detector are assembled in the chamber. To acquire the XPS spectra, Al Kα (*E* = 1486.7 eV) radiation was employed. The electron emission was recorded by setting the surface of the sample at an angle of 38° with respect to the detector. The binding energy was calibrated using the 1s peak signal from adventitious C at 284.6 eV.

The photoanodes were prepared by depositing a Co or Ni film, 30 nm thick, on Si/SiO_*x*,850_ and Si/SiO_*x*,RT_ substrates by DC magnetron sputtering (*P* = 50 W), using Ar as working gas (*p* = 5 × 10^–3^ mbar). The sample was at room temperature during the deposition at a distance of ∼5 cm from the 2″ Co target (purity > 99.9%). The deposition rate was 2 Å/s. The film thickness was measured by AFM. A thermal oxidation of the deposited films was performed in a tubular furnace for 2 h at 300 °C under an O_2_ flow (15 Nl/h). Boron-doped Si(100) substrate (p^+2^-Si, resistivity < 0.005 Ω/cm^2^) was used to prepare in a similar way a reference electrode (Si/Co_3_O_4_) to estimate the photovoltage of the Co_3_O_4_ photoanodes.

Other Si/SiO_*x*_/Co_3_O_4_ photoanodes were prepared from Si/SiO_*x*,RT_ and Si/SiO_*x*,850_ substrates by DC magnetron sputtering (*P* = 50 W) deposition in a reactive atmosphere, i.e., Ar (9.5 sccm) and O_2_ (1.4 sccm). The working pressure was kept at *p* = 3 × 10^–3^ mbar. The obtained Co–O films (30 nm thick) were then annealed at 300 °C in O_2_ (20 Nl/h) for 1 h.

Ohmic electrical contacts were obtained on the back side of the Si substrates by depositing a Cr adhesion layer plus a 100 nm thick Au film by magnetron sputtering. For solid-state electronic impedance measurements, a similar contact (size of a few mm^2^) was deposited on the nanopetal side of the samples.

The photoelectrochemical (PEC) and electrical measurements were performed by using the Autolab PGSTAT204 instrument. All PEC measurements were carried out in 50 mL of a 1 M KOH solution at room temperature. Ag/AgCl (*E*° = 0.232 vs SHE, as measured before and after the PEC experiment) and Pt wire were used as reference and counter electrodes, respectively. The samples were mounted on a Teflon PEC cell (Pine Research Instrumentation) using an O-ring (area = 0.2826 cm^2^, used for the calculation of the current density) and using a Au tip in contact with the Au film deposited on the back side of the photoanode. The photoanodes were front-illuminated by AM 1.5G simulated solar light (sun simulator from LOT—Quantum Design, 100 mW/cm^2^ at the sample position) or by a white LED, (Philips LUMILEDS, *P* = 25 mW/cm^2^ emission spectrum in Figure S11), as specified in the text, through a quartz window. EIS measurements were performed in the frequency range 10^6^ to 0.1 Hz with an amplitude oscillation of ±10 mV. Linear sweep voltammetry curves were measured with a scan rate of 5 mV/s, while cyclic voltammetry curves were measured at 50 mV/s. LSV data were corrected by considering the voltage drop due to the internal resistance, which was measured by the high-frequency intercept of the impedance in the real axis in the Nyquist plot. A homemade setup provided with Au tips and connected to the potentiostat was used to perform solid-state electrical measurements.

Incident photon-to-current conversion efficiency measurements were performed using as a tunable monochromatic light source a SPEX’s FluoroMax spectrofluorometer provided with a Xe lamp. The current flowing in the photoelectrochemical cell was measured in chronoamperometry mode by illuminating the sample at a selected wavelength (bandwidth = 10 nm) from 350 to 770 nm, every 10 nm. The light intensity was measured by a Si photodiode (FDS100).

## Supporting Information Available

Additional characterizations, including SEM, AFM, GIXRD, UV–vis, XPS, and (photo)electrochemical analysis (PDF)
