# Supplementary material for: Effect of Interfacial SiOx Defects on the Functional Properties of Si-Transition Metal Oxide Photoanodes for Water Splitting
Source: ACS Appl Mater Interfaces. 2023 Oct 2;15(40):46933–40. doi: 10.1021/acsami.3c09555 (PMC10571009; doi:10.1021/acsami.3c09555)
Supplement: Supplementary file 1 — am3c09555_si_001.pdf [file am3c09555_si_001.pdf]

Effect of interfacial SiO_x defects on the functional properties of Si-Transition Metal Oxide photoanodes for water splitting
Supporting information

P. Ragonese,[†] B. Kalinic,[†] L. Franco,[‡] L. Girardi,[‡] B.M. Fernández Peréz,[†] D. Carbonera,[‡] G. Mattei,[†] G.-A. Rizzi,[‡] and C. Maurizio[*,†]

†*Physics and Astronomy Department, University of Padova, via Marzolo 8, I-35131 Padova, Italy*

‡*Department of Chemical Sciences, University of Padova, via Marzolo 1, I-35131 Padova, Italy*

E-mail: chiara.maurizio@unipd.it

Phone: +39 049 8277002. Fax: +39 049 8277003

September 19, 2023

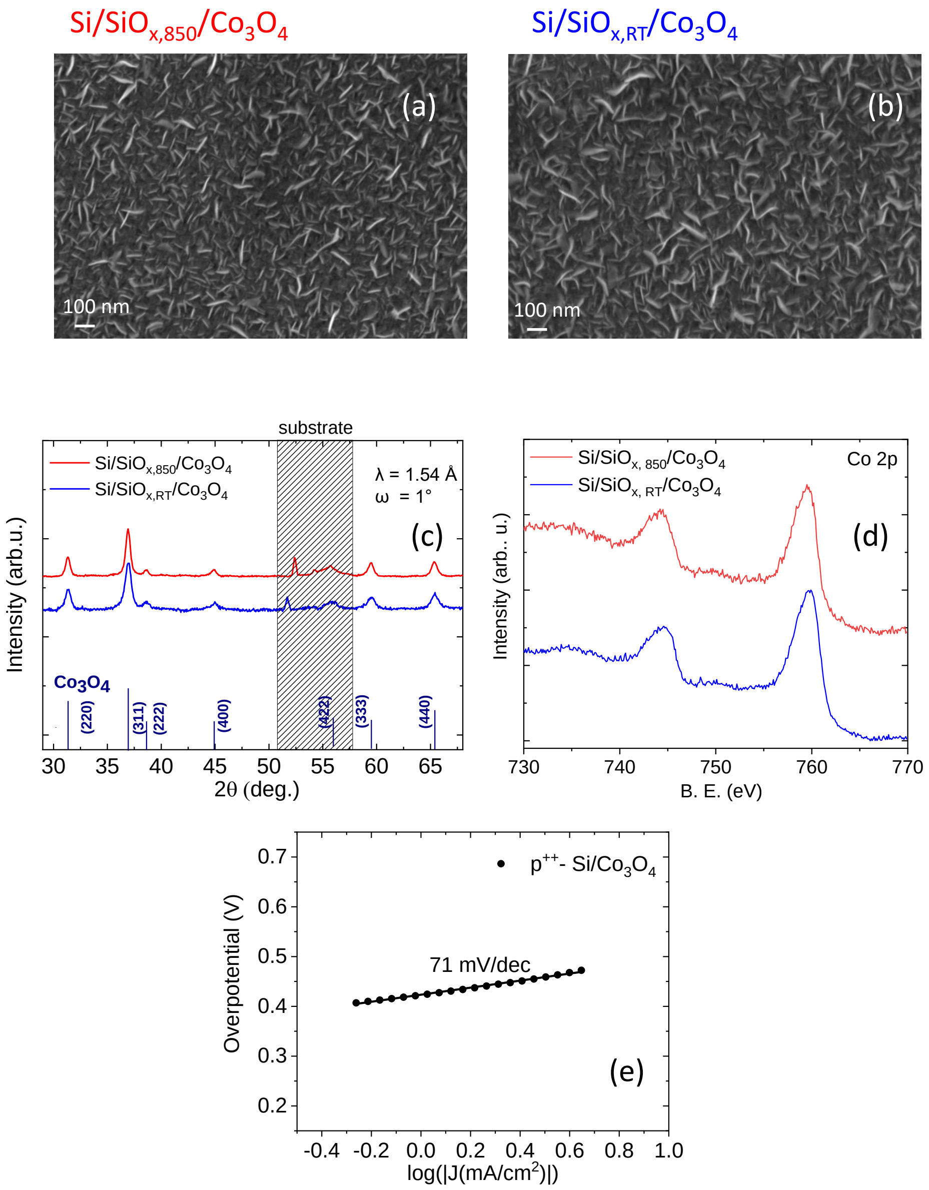

Figure S 1: (a,b) SEM images of Si/$SiO_{x,850}$/Co_3O_4 and Si/$SiO_{x,RT}$/Co_3O_4. (c) Grazing incidence X-ray Diffraction pattern (incident angle $\omega = 1°$) of Si/$SiO_{x,850}$/Co_3O_4 and Si/$SiO_{x,RT}$/Co_3O_4 photoanodes (spectra vertically shifted for clarity). The only crystalline phase is Co_3O_4, the average crystal size (Debye Scherrer analysis) is about 25 nm in both cases. The spurious scattering contribution from the substrate is limited to the shaded part. (d) XPS spectra for the Si/$SiO_{x,850}$/Co_3O_4 and Si/$SiO_{x,RT}$/Co_3O_4 photoanodes in the binding energy region of Co 2p levels (spectra vertically shifted for clarity). The very same features typical of Co_3O_4 are present in both cases [9]. (e) Tafel plot of p^{++}- Si/Co_3O_4 measured in 1M KOH.

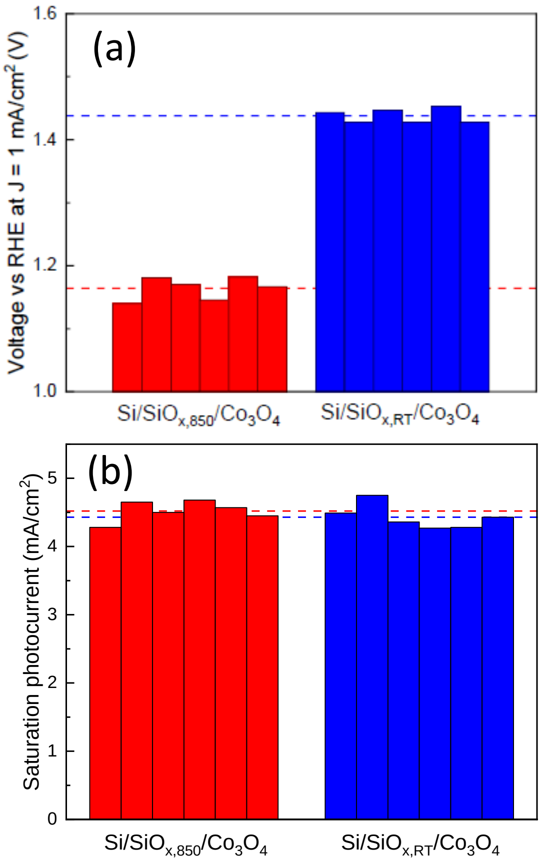

Figure S 2: (a) Voltage needed to develop a photocurrent of 1 mA/cm^2 when illuminated with a white led (25 mW/cm^2), and (b) saturation photocurrent for 6 different photoanodes of the kind Si/SiO$_{x,850}$/Co$_3$O$_4$ and 6 of the kind Si/SiO$_{x,RT}$/Co$_3$O$_4$. Figure (b) shows that the two kind of photoanodes exhibit the same saturation current.

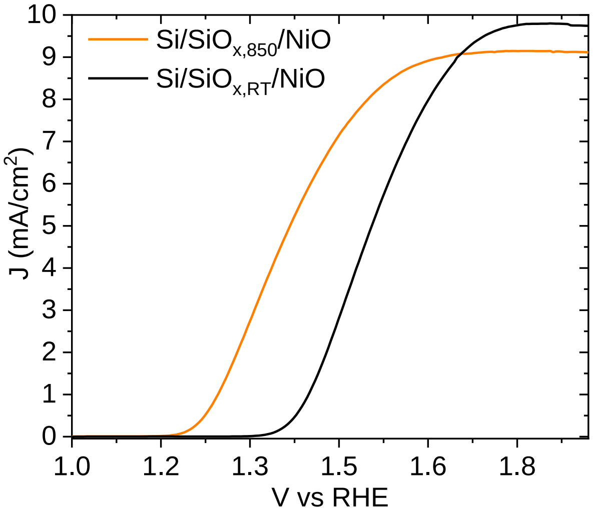

Figure S 3: Current-voltage curves recorded in 1M KOH under illumination with a white LED ($P = 25$ mW/cm^2) of Si/SiO$_x$/NiO photoanode, obtained by a deposition of a 35 nm-thick Ni film by magnetron sputtering on Si/SiO$_{x,850}$ and Si/SiO$_{x,RT}$ substrates, followed by thermal annealing in O_2 (20 Nl/h) for 2 h at 300°C.

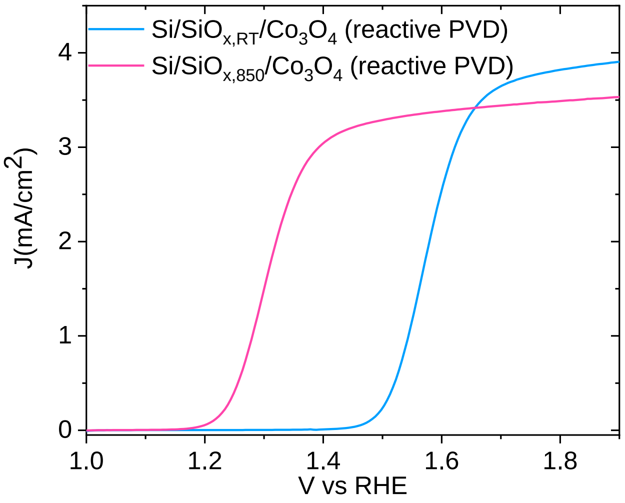

Figure S 4: Current-voltage curves recorded in 1M KOH under illumination with a white LED ($P = 25$ mW/cm^2) of Si/SiO$_x$/Co$_3$O$_4$ photoanodes obtained by reactive physical vapor deposition on Si/SiO$_{x,850}$ and Si/SiO$_{x,RT}$ substrates of Co in O_2-rich atmosphere, followed by thermal annealing at 300°C in O_2 (20 Nl/h) for 1h.

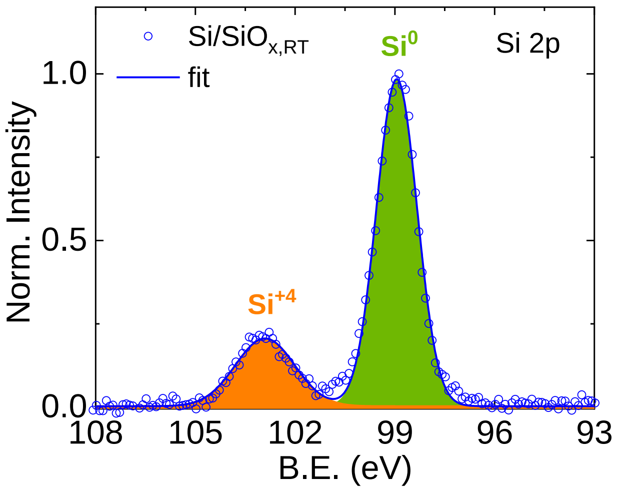

Figure S 5: XPS spectrum in the Si 2p electronic energy range for Si/SiO$_{x,RT}$ substrate and best fit curve obtained considering only the Si^0 (green) and Si^{+4} (orange) contributions.

	scr	qnr
J_0 (μA)	10.1 ± 0.1	1.64 ± 0.04
n	3.94 ± 0.01	2.60 ± 0.01

Figure S 6: Results of the linear fit (space charge region-scr and quasi-neutral region-qnr) of the solid state ln(J)-V curves measured for the Si/SiO$_{x,850}$/Co$_3$O$_4$ photoanode. Each region has been modelled according to the diode equation:

$$J = J_0(e^{\frac{e(V-r_sJ)}{nkT}} - 1)$$

where r$_s$ is the series resistance and n is the diode ideality factor.

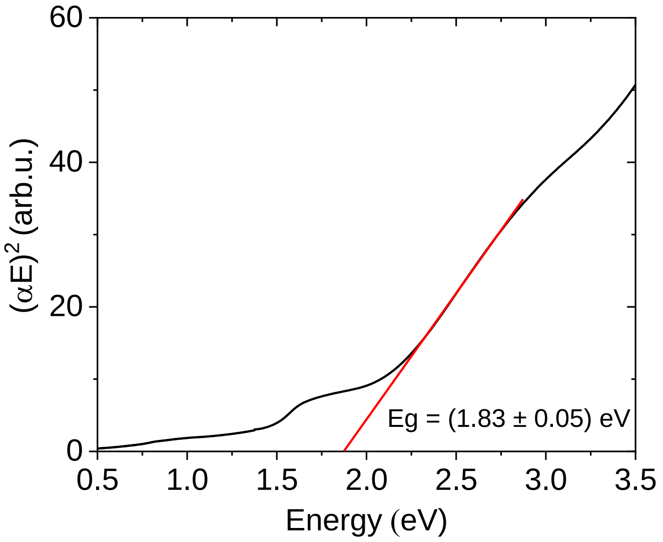

Figure S 7: Tauc plot of a Co_3O_4 nanopetals layer obtained by Co deposition (30 nm-thick) on a soda lime glass slide, annealed in O_2 for 2 h at 300°C.

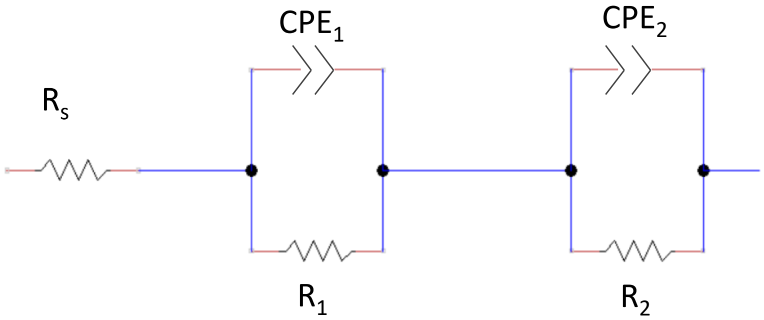

Figure S 8: Equivalent electrical circuit used for fitting the EIS data. Two Constant Phase Elements (CPE) are used, whose impedance is $Z_{CPE}=1/[C_{eff}\ (j\ \omega)^n]$. The two R-CPE loops in series describe the solid-electrolyte interface (R_1-CPE_1, resonant at low frequency) and the solid-solid interface (R_2-CPE_2, resonant at high frequency).

	$Si/SiO_{x,850}/Co_3O_4$	$Si/SiO_{x,RT}/Co_3O_4$
$R_s(\Omega)$	30	30
$R_1(\Omega)$	1.0×10^5	1.2×10^5
$C_{eff_1}(F)$	1.6×10^{-4}	1.8×10^{-4}
n_1	0.87	0.89
$R_2(\Omega)$	3.8×10^3	1.1×10^3
$C_{eff_2}(F)$	1.5×10^{-6}	1.7×10^{-7}
n_2	0.70	0.85

Figure S 9: Fitting results, according to the model circuit of Figure S8 of the EIS spectra recorded at the open circuit potential in 1M KOH in dark condition. Uncertainties are in the last digit.

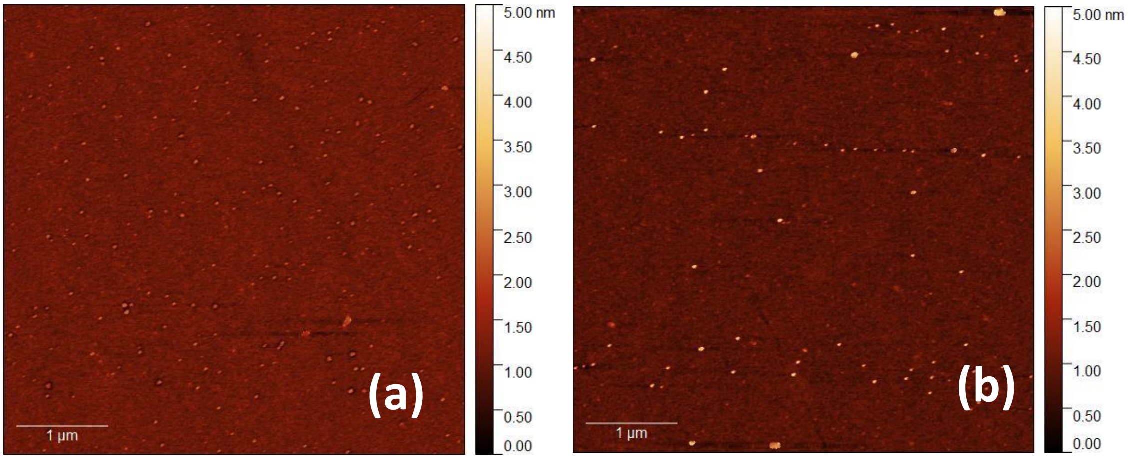

Figure S 10: AFM images of the $Si/SiO_{x,RT}$ and $Si/SiO_{x,850}$ substrates.

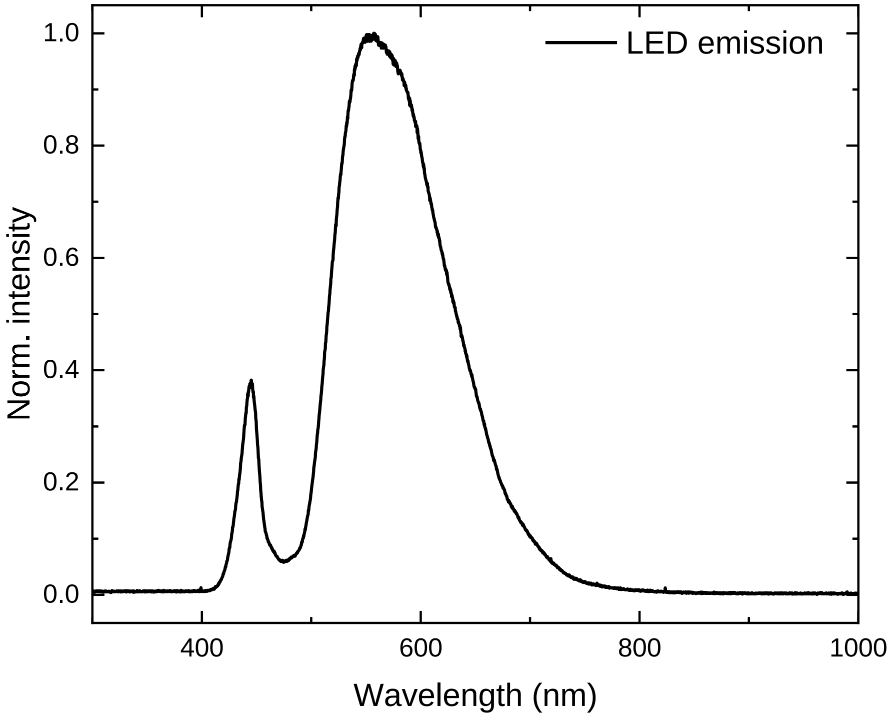

Figure S 11: Emission spectrum of the white LED.

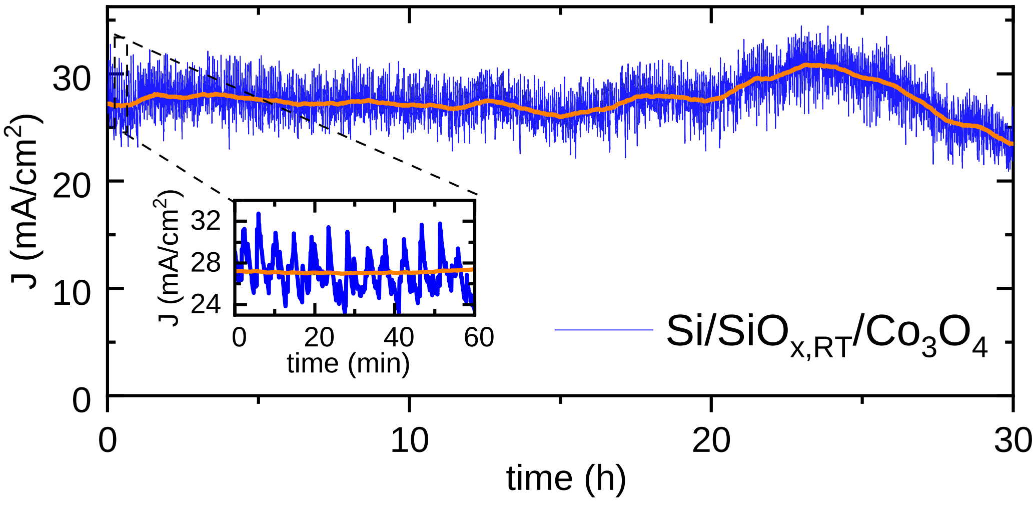

Figure S 12: Chronoamperometric curve measured in saturation condition for the Si/SiO$_{x,RT}$/Co_3O_4 photoanode. The inset shows the fluctuations of the current due to the evolution of bubbles.

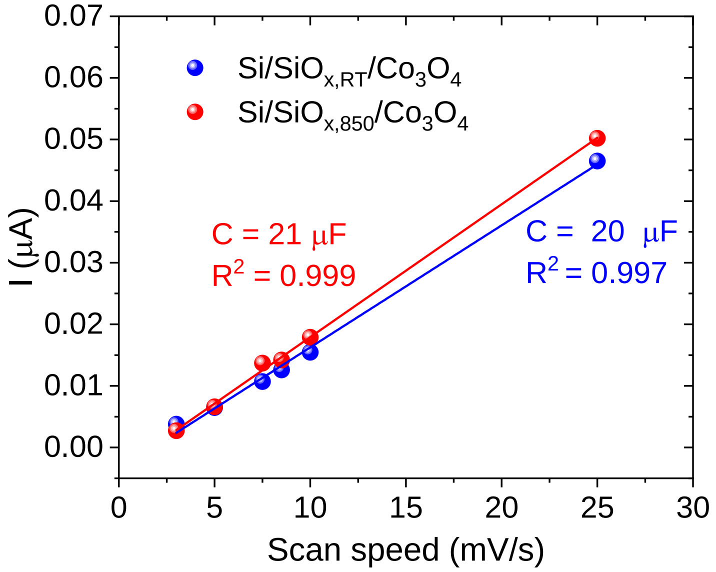

Figure S 13: Estimation of the electrochemically active surface area for the $Si/SiO_{x,850}/Co_3O_4$ and $Si/SiO_{x,RT}/Co_3O_4$ photoanodes, obtained by CV curves recorded at different scan rate. The solid lines are the best fit to the experimental data. The obtained values of the double layer capacitance are the same within the experimental uncertainty (5%), as expected. Considering that the geometric area is 0.2826 cm^2 and the reference value for a flat Co_3O_4 layer ($C = 20\ \mu F/cm^2$, from R. van de Krol, M. Gratzel, 'Photoelectrochemical hydrogen production', Springer, 2012), the electrochemically active surface area is ≈3.5 times the geometric one.
